# The transformative power of mHealth apps: empowering patients with obesity and diabetes – a narrative review

**DOI:** 10.25122/jml-2024-0340

**Published:** 2024-12

**Authors:** Alina Spinean, Simona Carniciu, Oana Alexandra Mladin, Cristian Serafinceanu

**Affiliations:** 1 Carol Davila University of Medicine and Pharmacy, Bucharest, Romania

**Keywords:** mobile health, mHealth, health apps, diabetes, obesity, behavior

## Abstract

The rising prevalence of obesity and diabetes underscores the need for innovative approaches to promote healthier lifestyles and improve clinical outcomes. Emerging evidence suggests that integrating mobile health (mHealth) technologies, such as smartphone applications and wearable devices, may provide a promising solution. mHealth interventions have the potential to enhance the delivery and accessibility of nutritional therapy and lifestyle modification programs for people with obesity and diabetes. This systematic review examines the available literature on the application and effectiveness of mHealth-based tools and technologies in managing these chronic conditions, offering insights into the current state of the field and opportunities for future research and clinical implementation. The review explores the diverse range of mHealth apps and devices utilized, their impact on weight loss, glycemic control, and other health-related outcomes, and the challenges and limitations associated with their use. This study highlights future directions and the growing importance of mHealth in the comprehensive management of obesity and diabetes to inform healthcare professionals, researchers, and policymakers about the potential of these innovative approaches to enhance patient care and improve population health.

## INTRODUCTION

The prevalence of chronic conditions, such as obesity and type 2 diabetes, has become a significant public health challenge worldwide, contributing to increased morbidity and mortality [1]. According to the World Health Organization (WHO), the prevalence of obesity has nearly tripled since 1975, while diabetes affects nowadays more than 537 million people globally and is predicted to rise to 783 million by 2045 [2,3]. The increasing prevalence of obesity and diabetes requires innovative approaches to dietary management and lifestyle intervention, as these conditions are often linked to unhealthy dietary patterns and sedentary lifestyles.

Traditional interventions of dietary management, including face-to-face consultations and printed materials, often face challenges and fall short in terms of adherence, long-term effectiveness, or access to healthcare resources. Mobile health applications (mHealth apps) offer a promising solution by leveraging technology to provide real-time patient support, education, and patient monitoring. The advent of mHealth applications presents a transformative opportunity to enhance patient engagement, facilitate better tracking of dietary intake, and ultimately promote healthier lifestyle choices [4]. Various dietary mobile applications are widely available and accessible to the public, and these apps are primarily used as effective tools for tracking food intake and physical activity [5]. The potential of digital interventions lies in their ability to reach a large population at a relatively low cost, making them a promising approach for promoting healthy lifestyle behaviors and managing chronic diseases [6].

This article reviews the current state of mHealth applications in nutritional therapy and lifestyle interventions for obese and diabetic patients, focusing on their mechanisms of action, outcomes, and the role of healthcare professionals in their implementation. This article explores the multifaceted role of mHealth apps in developing and implementing personalized nutrition plans, emphasizing their potential to improve food awareness, promote adherence to dietary guidelines, and foster lasting behavior changes, examining their effectiveness, user engagement, and the potential for improving health outcomes. We also discuss the technological advancements that facilitate personalized dietary recommendations and the implications for healthcare providers and patients.

### mHealth Apps: definition and functionality

mHealth apps are mobile applications that support various health-related activities, including disease management, health education, and self-monitoring [7]. These apps often include features such as food tracking with real-time feedback on dietary choices, personalized recommendations, physical activity monitoring, glycemic monitoring, water intake, weight monitoring, educational materials, and personalized feedback. By empowering users to log their food intake and physical activity, mHealth apps can foster awareness of dietary habits and encourage healthier choices [8].

Integrating diverse functionalities, mobile health applications can empower patients with chronic conditions to actively manage their health. These applications can provide access to online educational resources, facilitate connections with peer support communities, and offer comprehensive lifestyle monitoring tools to track dietary intake, physical activity, and other health-related behaviors [9]. By promoting self-engagement and self-compliance, mobile health applications can play a pivotal role in disease management, ultimately leading to improved health outcomes for people with obesity and diabetes [10].

Mobile health applications provide a cost-effective and personalized approach to managing chronic conditions like obesity and diabetes [1,10,11]. The widespread use of smartphones in daily life offers a unique chance to leverage these devices for healthcare, overcoming geographic barriers and enabling real-time access to medical support [12].

The transformative potential of mobile health applications is found in their capacity to empower patients, bolster self-management strategies, and bridge the divide between patients and healthcare providers [13]. As smartphone usage continues to proliferate, incorporating mobile health applications into chronic disease management approaches can reshape how people with obesity and diabetes engage with their healthcare, ultimately cultivating enhanced health outcomes and improved quality of life [14].

### mHealth apps and personalized nutrition plans

mHealth apps allow the customization of dietary plans based on individual needs, preferences, and medical conditions. Users can input their dietary restrictions, caloric goals, and nutritional requirements, enabling the app to generate tailored meal plans. Research indicates that personalized interventions can improve adherence to dietary recommendations, leading to better health outcomes [15,16]. Personalized nutrition, defined as dietary advice tailored to an individual's unique characteristics, has become vital to nutritional therapy. Research suggests that personalized nutrition plans delivered through mHealth apps can significantly improve health metrics. Studies have shown that users are more likely to achieve their dietary goals when provided with tailored recommendations, leading to better management of conditions such as obesity, diabetes, and heart disease [17,18].

mHealth applications use advanced algorithms and machine learning techniques to analyze user data, including dietary habits, biometric information, and lifestyle factors. This data-driven approach allows for the creation of personalized nutrition plans that align with an individual’s specific health goals, such as weight loss, management of chronic diseases, or enhancement of athletic performance [19].

The interactive nature of mHealth apps fosters user engagement, which is crucial for successfully implementing personalized nutrition plans [20]. People can receive personalized advice at their fingertips, empowering them to make informed dietary choices regardless of location. Features like goal setting, progress tracking, and reminders help users stay motivated and accountable. Gamification elements, such as rewards and challenges, further enhance user participation, making adherence to dietary recommendations more achievable.

### mHealth apps, food tracking, and awareness

This self-monitoring promotes awareness of eating habits and encourages healthier choices. Studies have shown that food tracking is associated with weight loss and improved glycemic control in patients with diabetes. A meta-analysis in *Health Psychology* found that users who regularly documented their food intake lost 1.5 times more weight than those who did not. By visualizing their consumption patterns, users can identify areas for improvement, leading to healthier eating behaviors [21].

Another study published in the *Journal of Nutrition Education and Behavior* [22] found that participants who tracked their food intake using mobile applications reported increased knowledge about portion sizes and nutrient content. Studies show that patients using mHealth apps demonstrate higher adherence to dietary recommendations. For instance, a randomized controlled trial found that participants using a food tracking app were more likely to follow a prescribed diet compared to those receiving traditional counseling alone.

### mHealth apps and patient engagement

Integrating mobile health applications into chronic disease management strategies can revolutionize patient engagement with healthcare. These apps are powerful tools, allowing users to track their exercise routines, receive medication reminders, and even be prompted to engage in physical activity, all through the convenience of their mobile devices. Additionally, mobile health apps provide patients access to online educational resources, informing them about their conditions and the latest treatment approaches. Furthermore, these apps facilitate the creation of virtual peer support communities, where people facing similar challenges can connect, share their experiences, and learn from one another's coping strategies [9]. By leveraging mobile health app capabilities, patients with chronic conditions like obesity and diabetes can adopt a more proactive and empowered approach to self-management, leading to improved adherence, better self-management, and enhanced health outcomes.

The widespread adoption of mobile health applications has the potential to revolutionize healthcare, particularly for people with chronic conditions such as obesity and diabetes. Leveraging the ubiquity of smartphones, these innovative tools can provide personalized, cost-effective, and easily accessible solutions that cater to the specific needs of patients, transforming the way they manage their chronic conditions [23].

### mHealth apps and behavior modification

Mobile health applications can cultivate lasting behavioral change in people with chronic diseases like obesity and diabetes. By furnishing personalized tools, educational materials, and virtual peer support networks, these innovative digital platforms can enable patients to assume a more proactive and self-guided approach to managing their health. This can culminate in enhanced treatment adherence, improved self-management strategies, and ultimately, elevated health outcomes and quality of life for those grappling with these chronic conditions [24,25]. mHealth apps can play a pivotal role in managing these chronic conditions by leveraging established behavioral change theories, such as the Health Belief Model, Social Cognitive Theory, and Transtheoretical Model.

The Health Belief Model posits that an individual's willingness to undertake health-promoting actions is influenced by their perceived susceptibility to the condition, severity, and perceived benefits of the behavior [15]. mHealth apps can leverage this framework by providing personalized risk assessments, highlighting the potential long-term consequences of the individual's current practices, and demonstrating the concrete advantages of adopting healthier habits through interactive goal setting, progress tracking, and motivational feedback [10].

The Social Cognitive Theory further underscores the critical role of self-efficacy, or an individual's belief in their ability to execute a specific behavior, in facilitating behavior change. mHealth apps can enhance self-efficacy by providing users with opportunities for mastery experiences, vicarious learning through peer modeling, and verbal persuasion through tailored encouragement and coaching [26]. According to the Social Cognitive Theory, an individual's behavior is shaped by personal characteristics and environmental influences. mHealth apps can leverage this theory to promote behavior change by enhancing users' self-efficacy, offering social support through peer-to-peer communities, and allowing people to observe and emulate the successful behaviors of other app users [26].

The Transtheoretical Model, also referred to as the Stages of Change framework, describes the different phases people go through when altering their behaviors, ranging from precontemplation to maintenance stages [8,26]. By aligning mHealth applications with these distinct stages, tailored interventions, educational resources, and reinforcement strategies can be provided to effectively guide users through the behavior change process.

Integrating these behavioral change theories in the design of mHealth apps has demonstrated promising results in improving dietary habits, increasing physical activity, and supporting weight management among obese and diabetic populations [8,25-28]. However, it is crucial to address the challenges of user engagement and long-term adherence to ensure the sustained effectiveness of these digital interventions [20].

Emerging research has highlighted the promise of incorporating well-established behavior change theories into the design and development of mHealth applications. This approach has demonstrated the potential to boost user engagement, improve adherence, and enhance the effectiveness of nutritional therapy and lifestyle interventions for people grappling with obesity and diabetes [1,17,28]. For instance, apps that offer real-time feedback, personalized goal-setting, and virtual coaching have been found to foster self-engagement and self-compliance, leading to improved health outcomes. By aligning the capabilities of mHealth apps with established behavior change models, healthcare providers can empower patients to take a more active and informed role in managing their health, thereby amplifying the effectiveness of these interventions [4,14,22].

By integrating established behavioral change theories into the design and development of mHealth apps, healthcare providers can enhance user engagement, improve adherence, and increase the effectiveness of nutrition and lifestyle interventions for people struggling with obesity and diabetes. These apps can leverage models like the Health Belief Model, Social Cognitive Theory, and Transtheoretical Model to personalize risk assessments, highlight the consequences of current behaviors, and showcase the benefits of healthier habits. This can foster self-efficacy, provide social support, and guide people through the stages of behavior change. Recent studies have demonstrated the potential of this approach, with apps offering real-time feedback, personalized goal-setting, and virtual coaching leading to improved health outcomes [11,20,25].

### mHealth apps and challenges in chronic disease management

Mobile health applications can potentially enhance the management of chronic conditions like obesity and diabetes by empowering patients to adopt a more proactive approach to their healthcare [20,27,28]. These digital tools can foster self-engagement and self-compliance through personalized tracking features, educational resources, and virtual support communities, ultimately leading to improved health outcomes [9]. Furthermore, mobile health apps can facilitate enhanced communication and collaboration between patients and healthcare providers, enabling more informed clinical decision-making and personalized interventions. As the field of mobile technology continues to evolve, the transformative potential of these applications in chronic disease management is poised to expand, presenting new opportunities to enhance the quality of life for those living with these conditions [14,29].

### mHealth app and healthcare

mHealth apps can also serve as a valuable resource for healthcare providers [14]. By allowing patients to share their food tracking data with clinicians, providers can gain insights into dietary habits and make more informed recommendations. This data-sharing capability fosters collaborative care, where patients and providers work together to achieve health goals [1].

The transformative potential of mobile health applications extends beyond empowering individual patients; these technologies also possess the capacity to bridge gaps in healthcare accessibility, particularly among underserved and minority populations [6]. Mobile technologies can reach people who may encounter barriers to traditional healthcare, such as geographical remoteness, socioeconomic hurdles, or cultural and linguistic differences. By furnishing real-time access to medical support and personalized guidance, mobile health apps can help mitigate disparities in healthcare delivery and ensure equitable access to resources for managing chronic conditions [6,12].

Integrating mobile health applications into clinical practice can alleviate the workload on healthcare providers. This allows them to concentrate on addressing more complex patient needs while leveraging the applications to monitor patient symptoms, track medication adherence, and facilitate streamlined communication with their patients [7,14].

Mobile health apps are crucial for addressing healthcare disparities as the population becomes more diverse. These apps empower patients, improve communication, and optimize healthcare delivery. This helps address the unique challenges faced by people with chronic conditions like obesity and diabetes, leading to better health outcomes for all [10].

To maximize the benefits of mHealth apps, it is essential to integrate them into clinical practice effectively. Healthcare providers can educate patients by providing training on using mHealth apps effectively, emphasizing their role in dietary management and lifestyle interventions. Physicians can also encourage data sharing, enable collaborative goal setting and personalized feedback, and monitor progress. Reviewing patients’ app usage and dietary logs during consultations can reinforce accountability and motivation. Based on this information, they can customize recommendations. App suggestions should be customized to align with each patient’s unique needs, preferences, and health goals, ensuring a personalized and effective approach to dietary management [14].

Collaborating with a multidisciplinary team, including dietitians, exercise physiologists, and behavioral psychologists, can enhance the quality and effectiveness of educational resources. This collaboration can ensure that content is evidence-based and comprehensive, addressing the multifaceted nature of obesity and diabetes management.

### mHealth apps use and other benefits

Mobile health applications can empower patients to take a more active and self-directed approach to managing their chronic conditions, such as obesity and diabetes. These apps provide personalized tools for tracking health behaviors, accessing educational resources, and connecting with supportive communities. By fostering self-engagement and self-compliance, mobile health apps can play a pivotal role in improving health outcomes for people with obesity and diabetes [1,23].

By capitalizing on the widespread availability of smartphones and the convenience of mobile technology, these applications can make the process of adopting and maintaining healthier lifestyles more accessible and engaging for patients, leading to enhanced long-term health outcomes [25].

Integrating mobile health applications into managing chronic diseases, such as obesity and diabetes, holds substantial promise [1,10]. These innovative digital tools can empower patients to assume a more proactive role in their healthcare, cultivate effective self-management strategies, and facilitate enhanced communication and collaboration between patients and healthcare providers [23,30]. As the field of mobile technology continues to evolve, the potential for mobile health apps to revolutionize the approach to chronic disease management will only continue to grow, presenting new and exciting opportunities to improve the lives of people living with these conditions.

### Challenges and limitations

Nonetheless, the successful implementation of mobile health technologies also presents distinct hurdles that require attention. A paramount challenge is ascertaining digital equity and accessibility, as it is vital to make these applications approachable and user-friendly for people from diverse socioeconomic and cultural milieus [31]. Furthermore, surmounting language and cultural barriers is essential to cater to the varied needs of underserved populations and cultivate widespread adoption.

A crucial limitation is the need for more advanced algorithms and artificial intelligence-powered features in mobile health applications. While existing apps provide personalized tracking and educational resources, implementing sophisticated AI-driven recommendations and real-time guidance could further enhance their ability to deliver tailored patient support [32,33]. However, this advancement necessitates a sustained collaboration between healthcare professionals and technology experts to ensure the integrity and effectiveness of these digital tools.

Ensuring the long-term viability and healthcare system integration of mobile health applications present significant challenges. Seamlessly integrating patient data from these apps with electronic health records and garnering the buy-in of healthcare providers are crucial steps to enable widespread and efficacious adoption of these technologies [4]. Addressing these limitations will be essential to fully realize the transformative potential of mobile health in chronic disease management and enhance the overall well-being of affected people.

## DISCUSSION

With mobile health apps increasingly being employed, it is necessary to look at diversity and inclusion when developing and utilizing such devices. There are people of many different backgrounds at risk of experiencing digital inequality, including those lacking in digital literacy and access capabilities due to class as well as other forms of marginalization. By considering these factors, mobile health applications can reduce the potential for inequities and be more effective across historically underserved and minority populations, contributing to their capacity to reduce health disparities and improve overall population health.

New trends in mobile health applications are promising and may help revolutionize the chronic care management of diseases such as obesity and diabetes [34]. As mobile technology advances, these are bound only to enhance patient-first healthcare at a higher level.

Mobile health applications can now provide digital medical suites, which researchers may seek to optimize over time using some of the currently available recommendations for mHealth technology [35]. Examples of these advancements can be the applications of more sophisticated algorithms, and AI features that could provide personalized patient recommendations and instantaneous guidance to patients based on their health requirements or preferences [7,33]. In addition, wearable devices incorporated with biometrics sensors may allow greater self-monitoring, leading to data-driven decision-making that further supports patients in managing their chronic conditions [36].

As mobile health apps are increasingly being adopted, the digital divide must be addressed to ensure these technologies remain accessible and inclusive [1]. Mobile health applications also have the potential to be truly transformational in addressing health disparities and population health by offering solutions that address the language, culture, and special needs of underserved populations [6,12].

In chronic disease management, the promise for mobile health is vast. In conclusion, digital health tools can empower patients with obesity and diabetes to manage their chronic conditions more effectively and improve their quality of life when developed with continuous innovation, with consumers directly using these applications in collaboration with healthcare professionals and technology experts [27,29].

Along with the pressing privacy and data security issues that need to be addressed, a number of technological challenges currently act as barriers to the scale level that would facilitate meaningful penetration of mHealth in the broader ecosystem. The large number of mHealth apps across different categories is a significant problem, making it harder for providers and patients to understand the quality and reliability of such digital tools [37].

The dawn of the mobile phone revolution and the subsequent development of multiple mobile health applications have provided major benefits to healthcare, especially personalized, convenient, real-time patient monitoring and management [38-40]. Unfortunately, these new technological capabilities come with some drawbacks, most notably in terms of data privacy and security.

Although the mHealth apps have many advantages, one of their disadvantages is how apps manage sensitive health data. Such apps often process a large amount of highly sensitive personal health data, making them attractive targets for cybersecurity threats and subjecting their users to potential privacy violations [41]. While there are regulations in place that govern how data is stored and processed, studies have found that a large number of mHealth apps do not meet these standards. As the mHealth industry expands its reach through an increasing number of apps, securing comprehensive protection for health data is more pressing than ever. Healthcare providers and patients share these legitimate fears of data abuse, theft, or unauthorized usage — breaches that would have a detrimental effect on individual privacy and overall trust in mHealth technologies.

However, technological hurdles still present barriers to adopting mHealth on a larger scale. A major prerequisite is the excessive number and heterogeneity of mHealth apps released, which can make it very hard for healthcare personnel or patients to accurately quantify the quality, reliability, and clinical effectiveness of these gadgets [42]. While mobile health applications offer numerous benefits to the healthcare sector—enabling personalized, real-time patient monitoring and management—these advancements are accompanied by critical challenges, particularly regarding data privacy and security [43,44].

## CONCLUSION

Mobile health apps have a great potential to integrate into the chronic disease management landscape for conditions like obesity and diabetes. These innovative tools can increase patient engagement, foster self-management strategies, and enhance communication and collaboration between patients and healthcare providers. As mobile technology continues to evolve, so does the potential for mHealth apps to transform the landscape of chronic disease care. It is an open landscape, and we have only read the first page of possible solutions that can make a dramatic difference in the lives of those with multiple chronic diseases.

Using mobile health apps to treat chronic diseases that demand regular monitoring, like obesity and diabetes, has profound potential for transforming patient-centric healthcare. These digital, innovative tools enable people to be more active in their care, develop healthy self-management strategies, and promote better communication and collaboration with clinicians. For those with these chronic conditions, mobile health apps can facilitate self-engagement and compliance by providing personalized tracking features, educational resources, and virtual support communities, ultimately leading to better health outcomes and quality of life. In addition to their ability to promote self-management, mHealth apps can overcome geographical barriers in healthcare, enabling patients from remote or underserved areas to access the care and support they need. As mobile health technology continues to evolve, the potential for these apps in chronic disease management will only expand. With advancements in real-time monitoring, data-driven insights, and personalized care options, mHealth apps can improve long-term survival and overall quality of life for individuals affected by chronic diseases.
